# Sensing local energetics to acutely regulate mitophagy in skeletal muscle

**DOI:** 10.3389/fcell.2022.987317

**Published:** 2022-08-29

**Authors:** Anna S. Nichenko, Kalyn S. Specht, Siobhan M. Craige, Joshua C. Drake

**Affiliations:** Department of Human Nutrition, Foods, and Exercise, Virginia Polytechnic Institute and State University, Blacksburg, VA, United States

**Keywords:** mitochondria, mitophagy, AMPK, energetic stress, reactive oxygen species

## Abstract

The energetic requirements of skeletal muscle to sustain movement, as during exercise, is met largely by mitochondria, which form an intricate, interconnected reticulum. Maintenance of a healthy mitochondrial reticulum is essential for skeletal muscle function, suggesting quality control pathways are spatially governed. Mitophagy, the process by which damaged and/or dysfunctional regions of the mitochondrial reticulum are removed and degraded, has emerged as an integral part of the molecular response to exercise. Upregulation of mitophagy in response to acute exercise is directly connected to energetic sensing mechanisms through AMPK. In this review, we discuss the connection of mitophagy to muscle energetics and how AMPK may spatially control mitophagy through multiple potential means.

## Introduction

Skeletal muscle comprises approximately 40% of body mass (Janssen IH et al., 1985) and produces the locomotive force required for activities of daily living, as well as peak physical performance. Prolonged muscle contraction, such as occurs during acute exercise, requires an increase in ATP production that can be as much as 100-fold higher from rest (Weibel and Hoppeler, 2005). This profound energy production required to sustain long periods of contraction, is met largely by mitochondria. In skeletal muscle, mitochondria form an intricate reticulum that extends along the length of the individual cells (myofibers) (Kirkwood SPM and Brooks, 1986; Glancy et al., 2015; Glancy et al., 2017; Bleck et al., 2018), which functions as a syncytium to produce energy across the reticulum to aid muscle function (Glancy et al., 2015; Glancy et al., 2017; Bleck et al., 2018; Ghosh et al., 2018). The energetic requirements of prolonged acute exercise imposed upon mitochondria initiate a cascade of events, collectively referred to as mitochondrial quality control, that extend well beyond the cessation of contraction and synergistically act to adapt mitochondria for future energetic demands (Drake et al., 2016). In response to acute exercise, new mitochondrial proteins and lipids are synthesized (biogenesis) and incorporated (fusion) following energetic stress in order to expand the reticulum and increase functional capacity for mitochondrial respiration to more effectively meet the energetic demands of future events (Holloszy, 1967; Ryan and Hoogenraad, 2007). Inversely, removal and degradation of damaged and/or energetically deficient regions of the mitochondrial reticulum through mitophagy has only recently been shown to play integral roles in both the acute response and chronic adaptation to exercise in skeletal muscle (He et al., 2012; Lira et al., 2013; Laker et al., 2017; Moore et al., 2019). Mitophagy, therefore, may be an avenue for the development of therapeutics to promote functional and metabolic health of skeletal muscle that would have implications in a host of pathologies, including aging, where declining skeletal muscle health is characteristic.

Mitophagy is multifaceted as it requires the synthesis of an autophagosome, incorporation of cargo, fusion with a lysosome (creating an autolysosome), and, finally, degradation of the aforementioned cargo. Autophagosomes and lysosomes are not inherently mitophagy-specific but only become so by their recruitment/localization and incorporation of mitochondrial components. When activated in response to energetic stress, such as exercise, degradation through mitophagy occurs in spatially distinct domains (Glancy et al., 2017; Laker et al., 2017; Drake and Yan, 2019; Drake et al., 2021), suggesting autophagosomes and lysosomes may be synthesized in the vicinity of the mitochondrial region identified for degradation. Recent discoveries have suggested that the spatial specificity of mitophagy may be governed through locally distinct mechanisms at mitochondria, possibly in response to the energetic microenvironment (Miyamoto et al., 2015; Glancy et al., 2017; Zong et al., 2019; Drake et al., 2021; Schmitt et al., 2022). In this review, we briefly summarize the production of cellular energy by mitochondria and the biochemical and molecular consequences of energetic stress, namely acute exercise, in relation to mechanisms that locally monitor mitochondrial energetics to promote mitophagy and identify unresolved questions.

### Mitochondrial energetic stress as an initiation for mitophagy

**FIGURE 1:**
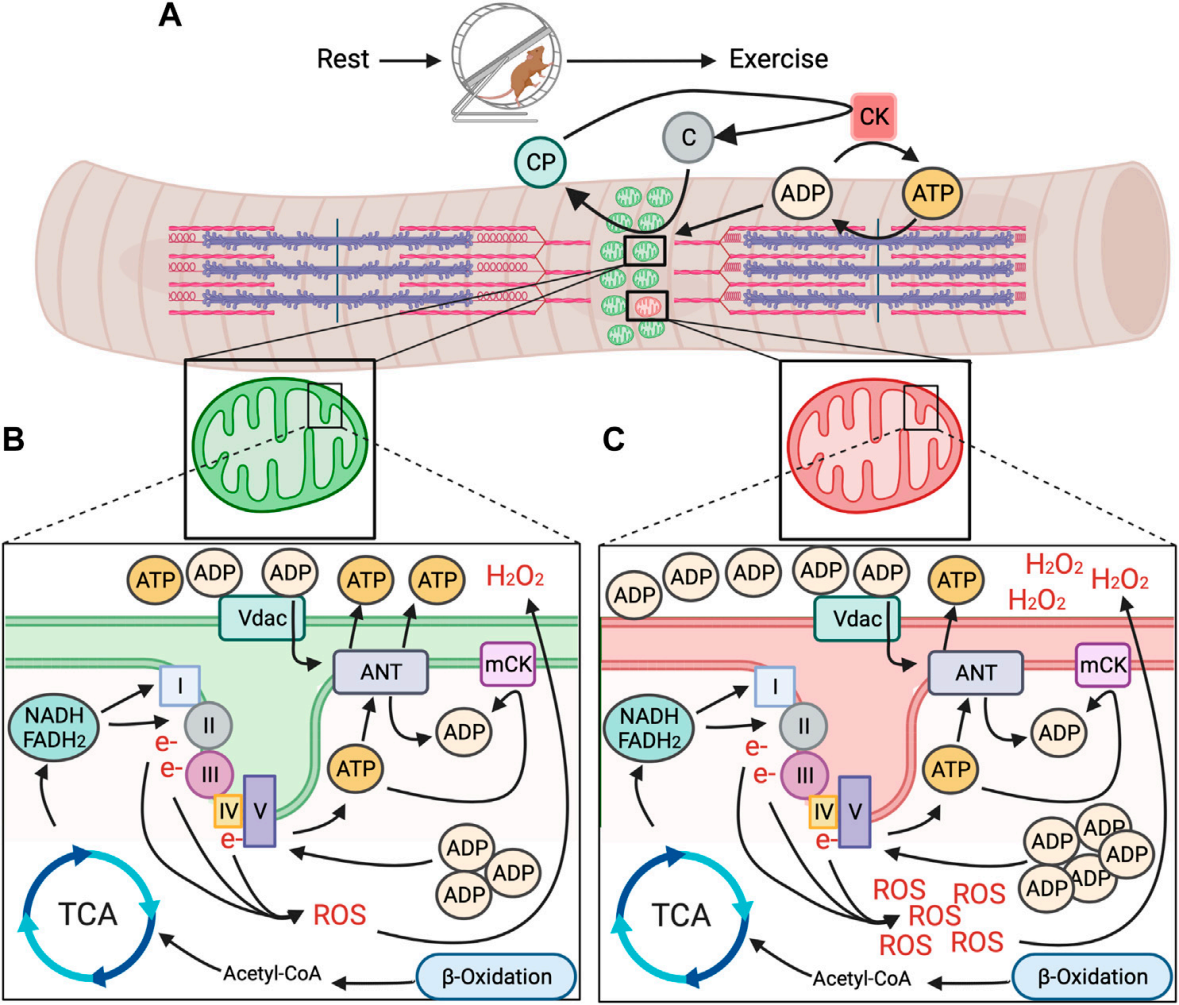
Model for mitochondrial energetic stress in skeletal muscle with exercise between healthy and unhealthy regions of the mitochondrial reticulum. **(A)**. Repeated muscle contraction with prolonged exercise requires sustained hydrolysis of ATP and donation of phosphates from creatine phosphate (CP), creating free creatine that is turned back to CP by mitochondrial creatine kinase (mCK) through the donation of a phosphate from intramitochondrial ATP. **(B)**. In healthy regions of the mitochondrial reticulum, accumulation of intramitochondrial ADP promotes generation of ATP from nutrient substrates *via* TCA cycle and *ß*-oxidation and through the ETC to meet energy demand, without undue accumulation in function altering intermediates (e.g., ROS). **(C)**. In spatially distinct unhealthy regions of the mitochondrial reticulum, ATP production does not keep pace with energetic demand, resulting in accumulation of intra and extramitochondrial ADP and excessive generation of ROS, potentially acting as energetic signals for removal *via* mitophagy.

Increased generation of energy in mitochondria is stimulated due to energetic changes that occur once muscle contraction begins (Figure 1). The contractile proteins actin and myosin hydrolyze ATP for contraction to occur (Figure 1A). The resulting ADP is rephosphorylated to ATP by cytoplasmic creatine kinase (CK) through donation of a phosphate from creatine phosphate (Wallimann et al., 1992; Piasecki et al., 2016). The subsequent free creatine diffuses across the outer mitochondrial membrane (OMM) and is rephosphorylated by mitochondrial creatine kinase (mCK) at the inner mitochondrial membrane (IMM), using intramitochondrial ATP as the phosphate donor. Additional influx of ADP *via* adenine nucleotide translocases (ANT) further increases intramitochondrial ADP (Bertholet et al., 2019). Increased extra- and intramitochondrial ADP levels is indicative of a loss in energetic homeostasis (i.e., energetic stress) and signals for generation of more ATP (Figure 1B).

Increased influx of ADP across the mitochondrial membranes promotes utilization of energetic intermediates from concurrent breakdown of nutrient substrates to generate ATP (Figure 1B). In general, these energetic intermediates come primarily from the breakdown of glucose and fatty acids. Metabolization of glucose (as well as lactate) and fatty acids during prolonged exercise results in pyruvate that is converted to Acetyl-CoA in mitochondria, the entry molecule to the tricarboxylic acid cycle (TCA) (van der Bliek et al., 2017). The TCA uses Acetyl-CoA to produce three molecules; ATP and the electron carriers nicotinamide adenine dinucleotide (Klionsky et al., 2016) and flavin adenine dinucleotide (FADH2). While ATP generated in the TCA cycle brings needed energy to the contracting muscle, NADH and FADH2 donate electrons to Complex I and II of the electron transport chain (CA et al., 2004; van der Bliek et al., 2017). These electrons are passed along to complexes with higher electron affinity, pumping protons into the intermembrane space, creating an electrochemical gradient (van der Bliek et al., 2017) and a store of potential energy. The flow of protons back across the membrane through the terminal Complex V (ATP synthase) converts accumulated intramitochondrial ADP due to muscle contraction back to ATP as it is pumped out into the cytosol.

Elevated AMP/ADP concentrations due to increased ATP hydrolysis during exercise causes activation of the conserved energy sensor, 5’ AMP-activated protein kinase (AMPK). AMPK is a heterotrimeric holoenzyme comprised of a catalytic ⍺ subunit and regulatory *ß* and *γ* subunits that exist in multiple isoforms encoded by distinct genes (e.g., PRKAA1 & PRKAA2 = α1 & α2, respectively; PRKAB1 & PRKAB2 = β1 & β2, respectively; PRKAG1, PRKAG2, and PRKAG3 = γ1, γ2, & γ3, respectively) (Ross et al., 2016a). In competition with ATP, AMP or ADP bind to the *γ* subunit of AMPK, causing a conformational shift that provides access to the T172 site on the *a* subunit for phosphorylation, fully activating AMPK, as occurs in response to prolonged exercise (Hawley SAD et al., 1996; Woods et al., 2003; Frøsig CJ et al., 2004; Hawley et al., 2005; Hurley et al., 2005; Shaw RJL et al., 2005). While AMPK is involved in a host of acute and chronic adaptive responses to exercise, AMPK directly phosphorylates the mitophagy initiating protein Unc 51 like autophagy activating kinase (Ulk1) at its activating phosphorylation site, S555 (Egan DFS et al., 2011; Bujak et al., 2015; Laker et al., 2017). When phosphorylated at S555, Ulk1 initiates formation of autophagosomes (Ganley et al., 2009) and knock-out of Ulk1 in skeletal muscle is sufficient to block exercise-induced mitophagy, as evidenced by the mitophagy reporter MitoTimer (Laker et al., 2014; Wilson et al., 2019), despite abundant lysosome recruitment (Laker et al., 2017). Alternatively, overexpression of a skeletal muscle-specific, dominant-negative form of AMPKα2 is sufficient to block exercise-induced mitophagy (Laker et al., 2017), in sum, connecting energy sensing of AMPK to mitophagy through Ulk1. As autophagy events are not observed at the sarcoplasmic reticulum post-exercise (Laker et al., 2017), the energetic stress experienced by mitochondria may be a stimulus for mitophagy *via* AMPK-mediated Ulk1 activation.

Interestingly, AMPK-Ulk1-mediated mitophagy in response to acute exercise occurs independent of other mitophagy regulators Pink1/Parkin (Drake et al., 2019; Seabright et al., 2020), suggesting AMPK-Ulk1 mitophagy axis as a uniquely energetic stress-dependent mechanism of upregulating mitophagy. This notion is supported by additional evidence of mitophagy in response to cardiac-ischemia being regulated through Ulk1 and independent of Parkin (Saito et al., 2019; Tong et al., 2021). However, the mechanism of exercise-mediated mitophagy may be related to exercise intensity and/or degree of energetic stress. Studies illustrating exercise-induced mitophagy through AMPK-Ulk1 signaling utilized a gradient intensity acute exercise paradigm with a fixed time frame (Laker et al., 2017; Drake et al., 2021). By contrast, in response to an exhaustive exercise paradigm, accumulation of the autophagosome membrane marker Lc3II is blunted in mitochondrial fractions isolated from skeletal muscle of Parkin knock out mice (Chen et al., 2018), suggesting Parkin may indeed have a role in exercise-induced mitophagy under certain energetic circumstances. Deletion of Parkin results in a number of mitochondrial as well as muscle defects (Gouspillou et al., 2018), which may cloud interpretation of its role in exercise-induced mitophagy. Parkin is an E3-ubiquitin ligase that accumulates on mitochondria in response to stabilization of Pink1 on the OMM due to a loss in membrane potential, accumulation in misfolded proteins, and/or mtDNA damage (Suen et al., 2010; Okatsu et al., 2012; Jin and Youle, 2013), none of which are associated with the acute exercise response (Fernstrom et al., 2004; Jafari et al., 2005; Wu et al., 2011). However, it is possible such stimuli occur in microdomains, and could be either a result or cause of local energetic dysfunction but future studies are needed to elucidate these possibilities.

**FIGURE 2:**
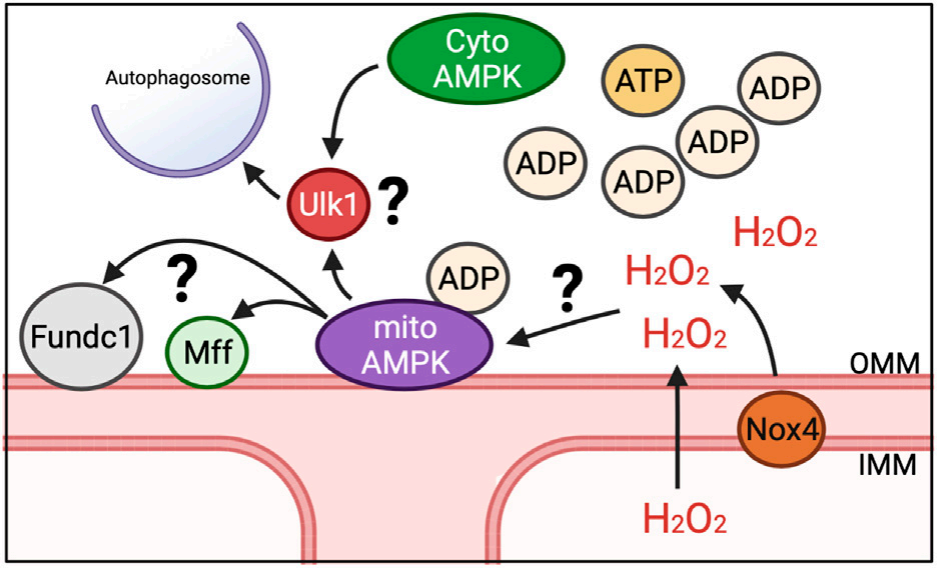
Potential mechanisms for induction of mitophagy in response to energetic stress. We have shown that mitoAMPK localizes to OMM and exerts control over exercise-induced mitophagy. Whether mitoAMPK coordinates with cytosolic pools of AMPK (cytoAMPK) to activate Ulk1 and initiate formation of autophagosomes is unknown. Additionally, it is unclear what roles other mitophagy-related proteins localized to mitochondria (e.g., Fundc1 and Mff) play in mitoAMPK regulation of mitochondrial quality control or the role of mitochondrial-generated ROS, potentially through Nox4.

While energy sensing of AMPK is connected to mitophagy through Ulk1 as a distinct mitophagy mechanism, it does not reconcile how energetic stress, such as exercise, would result in defined regions of the mitochondrial reticulum being spatially targeted for mitophagy. We and others have recently shown that AMPK localizes to mitochondria in multiple tissues (Zong et al., 2019; Drake et al., 2021), including skeletal muscle, where it is found on the OMM, which we have termed mitoAMPK (Drake et al., 2021). We were able to show that mitoAMPK activity could be selectively activated by inhibiting mitochondrial complex I activity in skeletal muscle *via* metformin (Drake et al., 2021), which would increase ADP concentrations in and around mitochondria (Foretz et al., 2010). *Via* fluorescent lifetime microscopy, we showed that mitoAMPK activity was particularly high in spatially distinct domains in cultured skeletal muscle myofibers following electrical stimulation-induced contractions (Drake et al., 2021), which is in agreement with the notion of overt mitochondrial energetic stress having some degree of spatial specificity. In addition, blocking mitoAMPK activity was sufficient to blunt exercise-induced mitophagy in skeletal muscle (Drake et al., 2021), linking local activation of mitoAMPK in response to energetic stress to mitophagy. Although, whether there is synergistic regulation of Ulk1 between mitoAMPK and other AMPK pools for mitophagy is unclear (Figure 2). In some cell culture systems, Ulk1 has been shown to localize to mitochondria (Wu et al., 2014; Tian et al., 2015), which is associated with mitophagy, but some evidence suggest that Ulk1 is dephosphorylated at S555 by the time it localizes to mitochondria (Hung et al., 2021). The continued advent of novel fluorescent activity reporters (Schmitt et al., 2022) will hopefully be able to shed new light on this area in the context of skeletal muscle and exercise.

Although AMPK is able to sense both AMP and ADP concentrations by their binding to the *γ* subunit of AMPK, emerging data suggests that ADP may be the predominant regulator in the context of exercise (Oakhill et al., 2012; Coccimiglio and Clarke, 2020). Skeletal muscle ADP concentrations during exercise are approximately 23 fold higher compared to AMP and parallel AMPK activity in relation to exercise intensity (Oakhill et al., 2012). Additionally, the high concentration of ADP during exercise is in excess of the dissociation constant for AMPK *γ* subunits (Oakhill et al., 2012), whereas AMP is not, further implying ADP may be more important. When considered in the context of the local accumulation of ADP both inside and outside of mitochondria during exercise, its plausible that local mitochondrial dysfunction would cause spatially distinct regions of high ADP concentrations (Figure 1C), which may serve as an energetic signal for AMPK activation and subsequent mitophagy. However, administration of the membrane permeable AMP-mimetic 5′-Aminoimidaxole-4-carboxamide ribonucleotide (AICAR) causes mitochondrial energetic stress and induces mitochondrial fission in an AMPK-dependent manner (Toyama EQH et al., 2016). This may suggest a scenario where energetic dysfunction in distinct domains could result in an additional phosphate being donated from ADP to generate ATP, causing a local increase in AMP. In skeletal muscle, mitoAMPK contains the γ1 isoform (Drake et al., 2021). AMP increases AMPK activity more than ADP *in vitro* when AMPK holoenzymes contain the γ1 isoform (Ross et al., 2016b). Thus, it is plausible that regions of distinctly high mitoAMPK activity in cultured myofibers following prolonged contraction (Drake et al., 2021) may be due to higher AMP vs. ADP concentrations in those areas of the mitochondrial reticulum and could be related to the spatial nature of mitophagy. However, whether such a spatial discrepancy between AMP and ADP occurs in skeletal muscle *in vivo*, and its relationship to mitochondrial quality control is unknown.

Other localized mechanisms of mitophagy on mitochondria, such as FUN14 domain containing 1 (Fundc1), is also shown to be dependent upon the AMPK-Ulk1 signaling cascade (Wu et al., 2014; Tian et al., 2015). While the functional role of Fundc1 in mitophagy is mostly understood in response to hypoxia *in vitro*, recent evidence showed that deletion of Fundc1 in skeletal muscle impairs exercise capacity and disrupts metabolism (Fu et al., 2018). Thus, Fundc1 could be a spatial partner with mitoAMPK to regulate mitochondrial quality through mitophagy in response to energetic stress (Figure 2). Additionally, BCL Interacting Protein 3 (BNIP3) localizes to the OMM and contains a docking site for the autophagosome (Marinkovic et al., 2021). BNIP3 expression increases with exercise training (Lira et al., 2013), suggesting that mitophagy capacity may increase. Whether BNIP3 is directly regulated by localized mechanisms, such as mitoAMPK or Fundc1, or passively used as a docking site is unclear. Additionally, mitochondrial fission, which is needed for select mitochondrial regions to be available for engulfment into autolysosomes but does not of itself necessitate that mitophagy will occur, is also upregulated with exercise (Moore et al., 2019). However, phosphorylation of the native cytosolically localized fission-related Dynamin-related protein 1 (Drp1) post-acute exercise is not impaired in mice over-expressing a dominant negative AMPKα2^11^ and inhibition of mitoAMPK activity does not impair pharmacological induction of mitochondrial fission in culture (Drake et al., 2021). Alternatively, mitochondrial fission factor (Mff), a mitochondrial localized fission protein, is an AMPK substrate (Toyama EQH et al., 2016), which may suggest that mitochondrial fission is coordinated between multiple-mechanisms that could be related to where mitophagy occurs or not. Future work is needed to elucidate how localized mechanisms response to energetic changes as well as elucidate additional substrates of mitoAMPK and their physiological roles.

### A role for reactive oxygen species in exercise-mediated mitophagy

Canonically, regions of mitochondria designated for degradation through mitophagy are “tagged,” typically by accumulation in ubiquitin that are recognized by autophagy receptor proteins, recruiting autophagosomes (Lazarou et al., 2015). However, what that signal may be in the context of exercise-induced mitophagy and how (or if) it coordinates with mitoAMPK activation is unknown. One possibility that may govern local tagging of mitochondria for mitophagy is a coordinated response to energetic stress through the localized production of reactive oxygen species (ROS). ROS are produced, in part, through the transfer of electrons along the ETC (Complex I and Complex III, though Complex II may produce ROS as well) (Figures 1B,C), as a byproduct of the TCA cycle and beta-oxidation, as well as by enzymes such as NADPH oxidases (Nox). ROS are highly reactive molecules that are able to damage biologic macromolecules such as lipids, proteins, and nucleic acids. While ROS can take many forms, the predominant forms produced in energetic stress are superoxide (O_2_^•-^) and hydrogen peroxide (H_2_O_2_). Superoxide cannot cross membranes and rapidly dismutates both spontaneously and enzymatically to H_2_O_2_ (Forman and Fridovich, 1973). However, H_2_O_2_ is membrane permeable and more stable, and affects proteins by reacting with cysteine (Cys) residues in proteins, resulting in altered activity and/or conformation (Sies and Jones, 2020).

The first observation that ROS may play an orchestrated role in mitophagy was in 2006 (Kissova et al., 2006). In yeast, it was found that rapamycin-induced autophagy was accompanied by early production of ROS and subsequent oxidation of mitochondrial lipids and blunting ROS production impaired the autophagic response. In mammalian cells this observation was furthered to include a specific target of mitochondrial H_2_O_2_ (Atg4) during nutrient starvation induced autophagy (Scherz-Shouval et al., 2007). *In vitro*, H_2_O_2_ directly inactivated Atg4 through oxidation of Cys 81. This oxidation and subsequent autophagy localized to mitochondria. Other proteins required for autophagy contain Cys residues that may also be specifically oxidized by localized ROS production (Filomeni et al., 2010), and may be a mechanism for mitophagy coordination by ROS.

During exercise, however, less ROS are produced through the ETC compared to basal respiration in skeletal muscle (Powers et al., 2020). Alternatively, following the cessation of exercise, there is a transient increase in mitochondrial oxidative stress (Laker et al., 2017), presumably due to a disconnect between continued electron flux and reduced ADP levels in mitochondria once muscle contraction stops. Exercise-induced mitophagy is preceded and coincides with this increase in mitochondrial oxidation, as evidenced by the oxidative-sensitive reporter gene MitoTimer (Laker et al., 2017). Analogous findings of elevated H_2_O_2_ coinciding with skeletal muscle mitophagy have been noted in disuse-induced muscle atrophy (Yamashita et al., 2021). In differentiated C_2_C_12_ myotubes, H_2_O_2_-induced autophagy is blunted by mitochondrial-targeted antioxidants (Rahman et al., 2014), suggesting production of mitochondrial ROS is not merely coincidental with mitophagy in skeletal muscle. Indeed, antioxidant/anti-inflammatory cocktails decrease markers of autophagosome formation (ATG7 and LC3) (Arc-Chagnaud et al., 2020). However, a mechanistic link between exercise-induced ROS and mitophagy remains unelucidated.

In addition to ROS from ETC, there are other potential sources of mitochondrial ROS. NADPH oxidase 4 (Nox4) has also been shown to localize to mitochondria, potentially to the inner mitochondrial membrane (Block et al., 2009; Ago et al., 2010; Graham et al., 2010; Sakellariou et al., 2013; Shanmugasundaram et al., 2017). Nox4 is part of the Nox family of proteins whose primary function is to produce ROS using NADPH as an electron donor and molecular oxygen as an electron acceptor. However, Nox4 is unique in that it primarily produces H_2_O_2_ over O_2_^•-.^ (Nisimoto et al., 2014), thus allowing for it to easily effect localized signaling. Nox4 contains an ATP binding motif (walker-A); thus decreasing H_2_O_2_ production when ATP is bound but increasing H_2_O_2_ production in low energy states (Shanmugasundaram et al., 2017). Therefore, it is plausible for Nox4 to play an integral role in modulating localized mitochondrial responses to energetic stress, as in exercise conditions. Deletion of Nox4 results in impaired adaptation to exercise, which includes defects in mitochondrial metabolism (Brendel et al., 2020; Specht et al., 2021; Xirouchaki et al., 2021). Furthermore, *in vitro* evidence suggests Nox4 is needed for recruitment of the autophagosome during energy limited conditions (Sobhakumari et al., 2013; Sciarretta et al., 2014). In sum, Nox4 has the ability to regulate the localized production of ROS in an energetically sensitive manner that is integral for mitochondrial health and could be important for driving mitophagy.

Localized production of mitochondrial ROS may also contribute to mitophagy through mitoAMPK. Although AMPK is understood canonically to sense ADP and AMP levels (Xiao et al., 2007; Xiao et al., 2011), there is evidence that AMPK activity may also be regulated by ROS (Shao et al., 2014; Morales-Alamo and Calbet, 2016; Trewin et al., 2018). ROS have been shown to induce AMPK activation even in conditions where cellular energy remains constant (Quintero et al., 2006; Emerling et al., 2009; Zmijewski et al., 2010; Wu et al., 2012). As Nox4 can localize to mitochondria and integrates cellular energy status to modulate mitochondrial health, Nox4 could potentially be important in the activation of mitoAMPK in response to exercise (Figure 2). Although one study observed that AMPK is similarly activated after exercise in wild type mice and mice lacking Nox4 (Vogel et al., 2015), there have not been careful temporal and spatial investigations of this potential interaction. Thus, it is plausible then that the localized production of ROS by Nox4 or other ROS producers, possibly at the mitochondria, has a role both in modulating mitoAMPK activity and in coordinating the localization of mitophagy along the reticulum in skeletal muscle.

## Conclusion

It is becoming increasingly clear that mitophagy can be regulated by localized mechanisms in response to energetic stress, underscoring the importance of energetic surveillance for homeostasis. The discovery of localized AMPK pools, in particular that of mitoAMPK, has added to our understanding of how this important energetic sensor regulates mitochondrial health. However many questions remain to be answered: how are particular AMPK holoenzymes targeted to mitochondria and elsewhere? are there localized fluxes in ADP and ROS that are discrete? how is mitoAMPK (and other localized pools) affected by disease? While recent observations have revealed exciting possibilities for energetic stress signaling, future directions will need to identify the temporal and spatial nature of these fluxes in response to energetic stressors such as exercise to truly tease out the physiologic importance of mitophagy and mitochondrial quality control in health and disease.
